# Influence of *CYP3A5* polymorphism on tacrolimus drug dosing in Indian renal allograft recipients: initial experience

**DOI:** 10.1186/1755-8166-7-S1-P98

**Published:** 2014-01-21

**Authors:** Mohan Patel, Manoj Gumber, Vivek Kute, Pankaj Shah, Himanushu Patel, Hargovind Trivedi, Aruna Vanikar, Jayesh Sheth

**Affiliations:** 1 Department of Nephrology and Clinical Transplantation, Institute of Kidney Diseases and Research Center, Dr HL Trivedi Institute of Transplantation Sciences, Ahmedabad, India; 2 Department of Pathology and Immunohematology, IKDRC-ITS, Ahmedabad, India; 3 Foundation for Research in Genetics and Endocrinology (FRIGE) Institute Of Human Genetics, Ahmedabad, India

## Background

Tacrolimus (Tac) is the mainstay of standard immunosuppressive regime in renal transplantation today. However, it needs regular drug level monitoring in view of narrow therapeutic window to keep blood level in therapeutic range. The objective of the study was to assess the potential influence of a functional polymorphism in CYP3A5*3 gene on dose requirements and trough blood levels of tacrolimus in renal transplant patients

## Materials & methods

This prospective observational study included 20 patients of end stage renal disease who underwent renal transplantion and a follow up of 1 year at our hospital. All the patients were started on standard immunosuppressive regime of Tacrolimus-Mycophenolate mofetil along with steroids with a starting dose of Tac 0.08 mg/kg/day. Genotype of *CYP3A5* was studied in 20 patients requiring low dose of Tac. At 7^th^ and 30^th^ day, and 3 monthly after transplant, Tac dosages (mg/kg/d), were adjusted based on blood levels and complications. Polymerase chain reaction, followed by restriction fragment length polymorphism analysis was used for genotyping CYP3A5*3 gene (A6986G) in intron 3.

## Results

Out of 20 patients (17 males and 3 females) included in the study, 16 had live related renal transplantation and 4 had diseased donor renal transplantation. Out of 20 patients 16 were found to have *CYP3A5* homozygous status (A3986G polymorphism in CYP3A5*3 gene-wild allele) and 4 with heterozygous status. Patients of *CYP3A5* homozygous status had mean Tac level of 16.84 ng/dL, median of 15.5 ng/dL (range- 13.2-25 ng/dL) on 7^th^ day of transplant. So Tac dose was reduced to achieve TDL and mean Tac level at 30^th^ day of transplant was 6.6 ng/dL and median of 7.4 ng/dL (range- 5.5-12.5 ng/dL). At 6^th^ month and 12^th^ month of transplant, mean Tac levels were 6.1 ng/dL and 5.9 ng/dL. In patients having *CYP3A5* homozygous status, mean and median Tac dose after 4 weeks was 0.03 mg/kg/day (range- 0.008 to 0.05 mg/kg/day) and mean creatinine was 1.17 mg/dL (range-0.8 to 1.4 mg/dL) after 30^th^ day. Remaining 4 patients having *CYP3A5* heterozygous status were maintaining TDL at Tac dose of 0.06 mg/kg/day. Five patients had graft biopsy during first 4 weeks of renal transplant showing acute tubular necrosis (ATN) in three patients, acute cellular rejection in one patient and cellular rejection with evidence of calcineurine inhibitor (CNI) toxicity in one patient.

## Conclusion

This study demonstrates the usefulness of *CYP3A5* genotype in transplant patients taking Tacrolimus-Mycophenolate mofetil and also shows that majority of our patients carry mutant allele A3986G in CYP3A5*3 gene.

